# Sensitivity of chemical weathering and dissolved carbon dynamics to hydrological conditions in a typical karst river

**DOI:** 10.1038/srep42944

**Published:** 2017-02-21

**Authors:** Jun Zhong, Si-liang Li, Faxiang Tao, Fujun Yue, Cong-Qiang Liu

**Affiliations:** 1 The State Key Laboratory of Environmental Geochemistry, Institute of Geochemistry, Chinese Academy of Sciences, Guiyang 550081, China; 2 University of Chinese Academy of Sciences, Beijing 100049, China; 3 Institute of Surface-Earth System Science, Tianjin University, Tianjin 300072, China; 4 State Key laboratory of Hydraulic Engineering Simulation and Safety, Tianjin University, Tianjin 300072, China

## Abstract

To better understand the mechanisms that hydrological conditions control chemical weathering and carbon dynamics in the large rivers, we investigated hydrochemistry and carbon isotopic compositions of dissolved inorganic carbon (DIC) based on high-frequency sampling in the Wujiang River draining the carbonate area in southwestern China. Concentrations of major dissolved solute do not strictly follow the dilution process with increasing discharge, and biogeochemical processes lead to variability in the concentration-discharge relationships. Temporal variations of dissolved solutes are closely related to weathering characteristics and hydrological conditions in the rainy seasons. The concentrations of dissolved carbon and the carbon isotopic compositions vary with discharge changes, suggesting that hydrological conditions and biogeochemical processes control dissolved carbon dynamics. Biological CO_2_ discharge and intense carbonate weathering by soil CO_2_ should be responsible for the carbon variability under various hydrological conditions during the high-flow season. The concentration of DIC_bio_ (DIC from biological sources) derived from a mixing model increases with increasing discharge, indicating that DIC_bio_ influx is the main driver of the chemostatic behaviors of riverine DIC in this typical karst river. The study highlights the sensitivity of chemical weathering and carbon dynamics to hydrological conditions in the riverine system.

Rivers play a crucial role in biogeochemical processes involved in the global carbon cycle, which link major carbon reservoirs, including atmosphere, biosphere, terrestrial geosphere, and oceans^1^. Chemical weathering is a fundamental geochemical process regulating the atmosphere-land-ocean fluxes and the climate on earth^2^,^3^. Forward and inverse models have been used for studying chemical weathering and CO_2_ consumption in many rivers around the world^4^,^5^,^6^,^7^. Many studies have focused on the chemical weathering and CO_2_ consumption in carbonate-rich catchments^8^,^9^,^10^, however, few of them have investigated the temporal dynamics of stream chemistry based on long term and high frequency sampling campaigns^11^, especially in carbonate-rich areas^12^,^13^. Carbonate weathering is highly sensitive to human activities and climate change^2^,^14^,^15^,^16^,^17^,^18^, the latter of which may partly be controlled by local hydrology^14^. Understanding the dynamics of the major dissolved solutes is useful for characterizing chemical weathering in rivers^11^,^16^. The feedback between hydrological conditions and chemical weathering is hypothesized to play a crucial role in modulating CO_2_ concentrations in carbonate-rich areas. Thus, time series observations are critical to better constrain the chemical fluxes, chemical weathering rates and the processes controlling the fate of chemical species in rivers^14^,^19^,^20^.

The annual fluvial carbon flux to the oceans is estimated to be 1 Pg C/y (0.8–1.2Pg C/y), of which 38% is in the form of dissolved inorganic carbon (DIC) and 25% is in the form of dissolved organic carbon (DOC)^21^,^22^, the rest is in the form of particulate carbon. The recognition that the carbon in inland rivers could be a substantial component in regional or global carbon budgets, has led to increased momentum in riverine biogeochemistry studies^23^. However, the patchy global estimates have been poorly constrained with respect to hydrological conditions, anthropogenic activities, acid rain etc.^22^,^24^,^25^,^26^. Riverine DIC and DOC concentrations are often, closely, associated with variations in hydrology^27^,^28^, as well as variations in sources and fluxes of dissolved carbon^26^,^29^. Detailed information on dissolved carbon dynamics with respect to hydrological conditions is still scarce, and relevant controlling processes are largely unknown. To constrain the DIC sources and clarify the relative biogeochemical processes, carbon isotopes can be used to identify the carbon sources and carbon biogeochemical processes^26^,^30^,^31^,^32^.

The continuous exposure of carbonate rocks on the Yunnan-Guizhou plateau is the largest karst area in the world^8^,^10^,^25^,^26^. The Wujiang River as one of the largest rivers in the Yunnan-Guizhou plateau is an ideal river to study the chemical weathering and carbon dynamics in the carbonate-rich areas^8^,^9^,^10^. A series of field campaigns have been conducted to sample river water under different hydrological conditions. This study uses data collected through the field campaigns to investigate correlations among chemical weathering, dissolved carbon dynamics, biogeochemical processes and hydrology. Carbon isotopes of dissolved inorganic carbon denoted as δ^13^C_DIC_, are used to identify carbon sources and constrain their contributions, which can provide insights into the main factors controlling carbon dynamics over time.

## Results

### Hydrochemical characteristics

pH of the river water samples is mildly alkaline (7.93–8.30) within the range of the whole Wujiang basin (7.6 to 8.9)^8^. The electrical conductivity (EC) values range from 315 to 407 μS/cm for the whole hydrological year (Table S1). Mean discharge-weighted concentrations can be calculated as Σ(*Q*_*i*_*C*_*i*_)/Σ*Q*_*i*_, where the subscript *i* represents each sample during the hydrological year^33^. The total dissolved solids (TDS) range from 239 to 356 mg/L (Table S1), with an average of 265 mg/L, which is higher than the world average value (97 mg/L)^22^. In comparison to rivers draining carbonate terrain worldwide, the average TDS value of the Wujiang River is higher than that of the South Han River (174 mg/L) in South Korea^34^, the Ganges and Indus rivers (164 mg/L) draining the Himalayas^35^,^36^ and the Mackenzie River (160 mg/L) draining the Rocky Mountains^37^. However, the TDS values are in the same range with that of the upper Yellow River (274 mg/L)^7^, the upper Xijiang River (297 mg/L)^25^ but lower than that of the Houzhai River (441 mg/L)^26^ in China. The total cationic charge (TZ^+^ = Na^+^+K^+^+2Ca^2+^+2Mg^2^, in μeq/L) and total dissolved anions (TZ^−^ = Cl^−^ + 2SO_4_^2−^ + HCO_3_^−^ + NO_3_^−^, in μeq/L) are well balanced, indicating that the unanalyzed ions play a minor role in charge balance. The mean discharge-weighted concentrations of major cations are as follows: Ca^2+^(1.33 mmol/L) > Mg^2+^(0.40 mmol/L) > Na^+^(0.17 mmol/L) > K^+^(0.04 mmol/L) (Fig. S1). The mean discharge-weighted concentrations of major anions are as follows: HCO_3_^−^ (2.37 mmol/L) > SO_4_^2−^ (0.44 mmol/L) > Cl^−^ (0.13 mmol/L) (Fig. S1). The Wujiang River shows a dominance of Ca^2+^, Mg^2+^, HCO_3_^−^ and SO_4_^2−^, which is similar to the characteristics of rivers draining karst areas^10^,^20^,^36^,^37^.

### Carbon characteristics

DIC is the sum of CO_2_ (aq), carbonic acid (H_2_CO_3_), HCO_3_^−^, and carbonate (CO_3_^2−^) ions^26^. The DIC concentrations in the river water vary from 2303 to 2783 μmol/L (Table S2), which is triple times higher than the world average concentration (852 μmol/L)^19^. The dissolved organic carbon (DOC) has a narrow range from 0.89 mg/L to 1.32 mg/L (Table S2), with no significant temporal variations. The partial pressure of carbon dioxide (*p*CO_2_) is a function of respiration, which can lead to increases in both riverine *p*CO_2_ and the dissolution of carbonate^26^. The *p*CO_2_ values range from 711 μatm to 1619 μatm (Table S2), which is much higher than the local atmospheric *p*CO_2_ (349 μatm) (Eq. S1). The value of δ^13^C_DIC_ ranges from −14.8‰ in the high-flow season to −9.4‰ in the low-flow season (Table S2), with the average value of −12.1‰.

### Temporal variations in major elements

The concentrations of major elements show significant temporal variations in the Wujiang River (Fig. S2). The discharge (Q) is low and relatively stable from November 2013 to March 2014 and relatively high from April 2014 to October 2014, reaching a maximum in July 2014 (Fig. S2). Generally, all the major elements (except Si) exhibit slightly decreasing trends during the high-flow season due to dilution and reach the maxima in February and March during the low-discharge period (Fig. S2). However, Si shows an increasing trend in the high-flow season relative to the low-flow season (Fig. S2).

### Relationship between elemental concentrations and discharge

Godsey *et al*.^33^ and Clow and Mast^38^ have demonstrated that the concentrations of weathering products are negatively correlated with discharge and can be approximated as power-law functions:


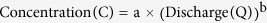


where a is a constant, and b represents the index that explains the deviation from chemostatic behavior^39^.

**Figure 1:**
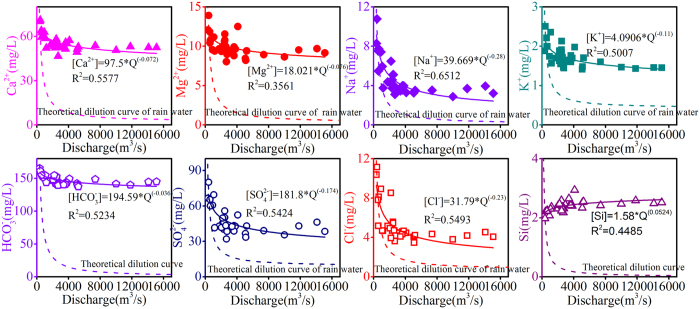
Concentration-discharge relationships of major elements (Ca^2+^, Mg^2+^, Na^+^, K^+^, HCO_3_^−^, SO_4_^2−^, Cl^−^, Si). The theoretical dilution curve means that these elements are diluted by deionized water (b = −1), and the theoretical dilution curve of rain water means that these elements are diluted by rain water, which is calculated by rainwater of Guiyang.

The regression coefficient b in the relationship between C and Q has a physical interpretation. If b = 0, the catchment behaves entire chemostatically; and if b = −1, Q is the only controller on C, constant solute fluxes being diluted by variable fluxes of water^27^. However, when b > 0, no dilution effect is present, because large amounts of inputs are induced by high discharge. Significant relationships (R^2^ values ranging from 0.35 to 0.65) between C and Q were found for the Wujiang River (Fig. 1). The slope values suggest that nearly all dissolved solutes (except Si) become diluted with increasing discharge and that the concentrations of Ca^2+^, Mg^2+^and HCO_3_^−^ do not vary as much as those of Na^+^, K^+^, SO_4_^2−^ and Cl^−^ (Fig. 1). Although all these dissolved solutes except Si decrease with increasing discharge, they do not strictly follow the theoretical dilution curve. Godsey *et al*.^33^ have postulated that concentrations are relatively constant in wide ranges of discharge, which maybe due to large amount of water stored in a catchment flows into the river induced by intense precipitation. Matrix porosity is widely distributed in the carbonate-rich areas, which stored large amount of “old” water. The near chemostatic behavior in the catchment may be ascribed to the carbonate-rich characteristics. The concentration of Si has a positive relationship with discharge (Fig. 1), indicating that Si is not affected by dilution, and that multiple biogeochemical processes counteract the dilution effect.

## Discussion

### Elemental ratio-discharge relationship

The dissolved loads of the river water are derived from atmosphere, rock weathering and anthropogenic pollutions^40^. The chemistry of river water in the Wujiang River is dominated by carbonate weathering (limestone and dolomite) (Fig. S3a). All of the samples have [Na^+^]/[Cl^−^] ratios exceeding 1 (Fig. S3b), indicating that silicate weathering is a clear source of major ions. There is no geological evidence for the exposure of evaporites in the river basin^8^, the contribution from evaporites is thus neglected. During carbonate weathering, the cations Ca^2+^and Mg^2+^are released into the dissolved phase. During silicate weathering, the cations Na^+^, K^+^, Ca^2+^ and Mg^2+^as well as Si, are released into the dissolved phase. Carbonate weathering is the primary sources of Ca^2+^, Mg^2+^ and HCO_3_^−^, and silicate weathering is the major source of Na^+^, K^+^ and Si in the basin^8^. Cl^−^ has two major sources: atmospheric and anthropogenic inputs^8^. Changes in DOC concentrations can be ascribed to mixing of multiple sources and biogeochemical processes^41^.

**Figure 2:**
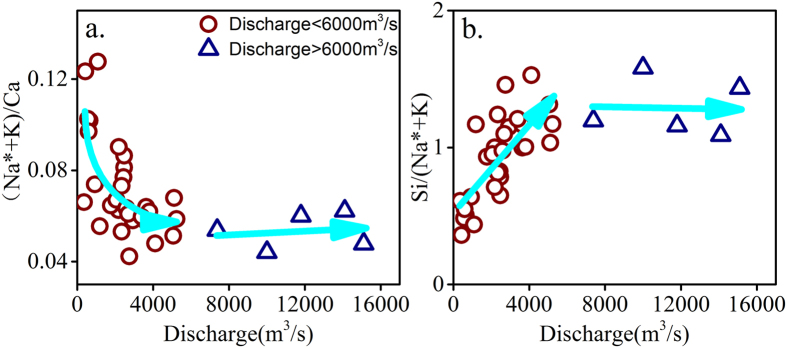
(**a**) Correlation between (Na* + K)/Ca ratio and discharge for the Wujiang River. (**b**) Correlation between Si/(Na* + K) ratio and discharge for the Wujiang River.

Elemental ratio-discharge relationships can be used to identify source changes and examine biogeochemical processes during various hydrological conditions^16^. Changes in elemental ratios are related to changes in the source or differential dissolution/precipitation rates between minerals, especially for carbonate and silicate minerals, with changing discharge^14^,^16^. As weathering progresses, saturation with respect to secondary silicate and the retention of silicate in the reservoir can buffer the concentration of dissolved Si, while the concentrations of dissolved cations that are not readily partitioned into secondary silicates continue to increase^16^,^42^. The variation in the ratio of (Na* + K)/Ca (Na* = [Na^+^] − [Cl^−^]) with changing discharge (Fig. 2a) suggests that the relative contribution of silicate mineral dissolution to the dissolved loads changes with discharge, which is similar to the findings of other studies^38^,^43^. Si, Na^+^ and K^+^ are from same lithologic source, and variations in Si/(Na* + K^+^) with discharge can be used to interpret the balance between secondary mineral precipitation and primary silicate weathering. The Si/(Na* + K) ratio can be regarded as a proxy commonly related to the “intensity” of silicate weathering^44^.

Because multiple concentration-discharge relationships occur with increasing discharge (Fig. 1), reactive transport can generate various behaviors in the ratio-discharge relationships. The observed variation in (Na* + K)/Ca with discharge in Wujiang River doesn’t show a linear positive relationship or negative relationship. As the mean discharge of the Wujiang River in the monsoon season is approximately 3000 m^3^/s, we define that discharge is two times greater than the mean discharge (i.e., 6000 m^3^/s) as extremely high discharge. The (Na* + K)/Ca ratio decreases rapidly with increasing discharge at discharge below 6000 m^3^/s (Fig. 2a). Thus, the slope of the (Na* + K)/Ca ratio-discharge relationship suggests that the relative proportion of solutes derived from silicate weathering decreases with increasing discharge at discharges below 6000 m^3^/s. However, (Na* + K)/Ca has a relative stable value with increasing discharge when the discharge is higher than 6000 m^3^/s (Fig. 2a). Under high discharge conditions with short fluid transit times, water cannot reach equilibrium with rocks^14^,^16^. With increasing discharge, carbonate minerals dissolve rapidly and can drastically alter the water chemistry composition relative to the silicate minerals^14^,^45^; thus, the (Na* + K)/Ca ratio decreases with increasing discharge. When discharge is greater than 6000 m^3^/s, as most of dissolved solutes concentrations have relative constant values with increasing discharge (Fig. 1), the relative proportion of solutes derived from silicate weathering versus carbonate has a narrow range.

The power law exponent describing the relationship between Si and discharge shows a positive trend (Fig. 1). Dissolved Si concentration is maintained by equilibrium with respect to secondary silicate mineral and the retention of silicate in the reservoir^38^,^42^,^43^. The Si/(Na* + K) ratio increases with increasing discharge for discharge below 6000 m^3^/s, suggesting that the relative release of Si to Na* + K from primary silicates is greater than lower discharge and less dissolved Si is retained in the reservoir with increasing discharge for discharges below 6000 m^3^/s. Increasing discharge, decreases the transit time of fluids, leading to less time for the fluids and minerals to reach equilibrium with a secondary Si-bearing phase, and less time for retention in the reservoir in the upper reach. Thus, the Si/(Na* + K) ratios increase with increasing discharge for discharge below 6000 m^3^/s. Extreme discharge will shift deep flow paths to fast near-surface flow paths. Because of the less transit time of water, there is less time for biogeochemical processes such as ions exchange, biological uptake. So the Si/(Na* + K) ratio is near to the ratio from silicate weathering at extremly high discharge. Therefore, samples show relative stable Si/(Na* + K) ratios with increasing discharge when the discharge is higher than 6000 m^3^/s.

### Chemical weathering fluxes affected by hydrological conditions

The temporal variability in discharge on the Yunnan-Guizhou Plateau in southwestern China mainly depends on rainfall associated with the monsoon climate. As discussed above, the major element dynamics are dominated by discharge, and hydrologic flushing further induces chemostatic behavior by increasing the reactive mineral surface area, which accelerates the mineral weathering^38^. In this study, a forward model is used to constrain the elemental sources (Eqs S1–9). Carbonate weathering fluxes (F_Carb_) and silicate weathering fluxes (F_Sil_) are calculated using Eqs S11 and 12, respectively.

**Figure 3:**
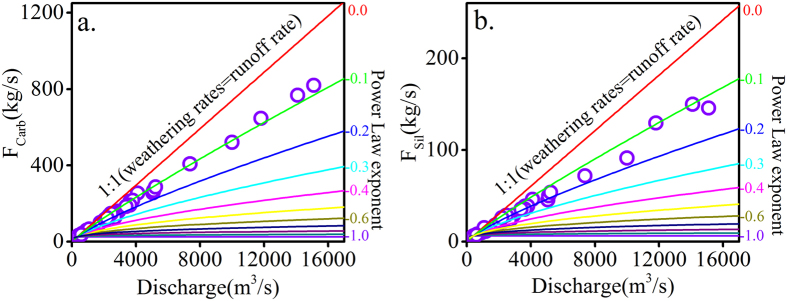
(**a**) Relationship between carbonate weathering fluxes (F_Carb_) and discharge. (**b**) Relationship between silicate weathering fluxes (F_Sil_) and discharge.

The results show a broad range: F_Carb_ ranges from 26.1 kg/s to 819.7 kg/s, F_Sil_ varies from 3.7 kg/s to 149.8 kg/s. F_Carb_ and F_Sil_ have strong correlations with discharge (Fig. 3a and b). The strong correlations between chemical weathering fluxes and discharge indicate that chemical weathering is dominated by hydrological conditions. Contours of different power law exponents spanning from dilution (−1) to “chemostasis” (0) (Fig. 3a and b) illustrate the sensitivity of chemical weathering to discharge changes. The power law exponent between F_Carb_ and discharge is approximately −0.1. F_Sil_ has a power law exponent of approximately −0.2 at relatively low discharge rates (e.g., discharge of <6000 m^3^/s) and approximately −0.1 at extremely high discharge rates (e.g., discharge of >6000 m^3^/s). Both F_Carb_ and F_Sil_ shows strong chemostatic responses to varying discharge, and F_Carb_ shows a slightly more obvious chemostatic response. The near chemostatic behavior of chemical weathering fluxes responding to changing discharge may be ascribed to the hypothesis that fluids will quickly approach chemical equilibrium in rapidly eroding environments^16^,^43^,^46^. Variations in chemical weathering fluxes versus discharge can be ascribed to the dissolution kinetics, because the dominance of physical erosion during high discharge allows the more easily weatherable carbonate to dissolve^14^. And Importantly, our results characterize the sensitivity of chemical weathering fluxes to changes in discharge.

### Response of dissolved carbon dynamics to hydrological changes

**Figure 4:**
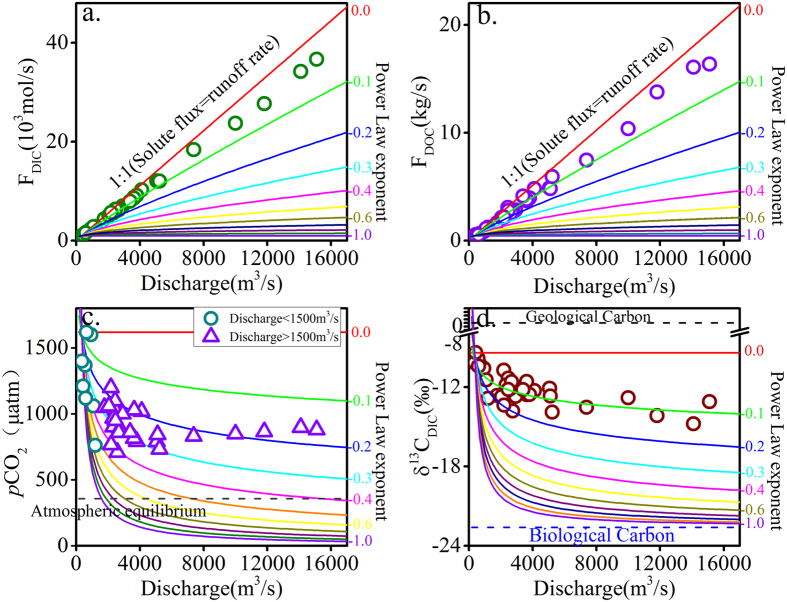
(**a**) Relationship between flux of DIC (F_DIC_) with discharge. (**b**) Correlation between flux of DOC (F_DOC_) with discharge. (**c**) Links between *p*CO_2_ and discharge. (**d**) δ^13^C_DIC_ values versus increasing discharge.

DIC is an important part of the total fluvial carbon to the ocean^47^. DOC is a significant constituent in aquatic ecosystems, and its concentrations in streams is influenced by both temperature and water flow pathway dynamics associated with changes in discharge^48^. The fluxes of DIC (F_DIC_) and DOC (F_DOC_) in rivers are strongly linked to climate condition (Fig. 4a and b). As discussed above, DIC and DOC show strong chemostic response with respect to varying discharge. Both F_DIC_ and F_DOC_ have strong positive relationship with discharge (Fig. 4a and b). The chemostic behaviors of DIC should be ascribed to primary production in the basin, as well as dissolution and precipitation. The power law exponents of both F_DIC_ versus discharge and F_DOC_ versus discharge are close to 0, indicating that the fluxes of dissolved carbon in the Wujiang River are dominated by hydrological conditions, and fluxes of DIC and DOC are sensitive to hydrological variability.

**Figure 5:**
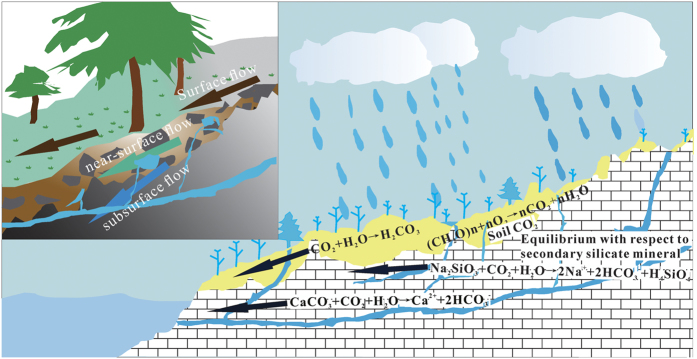
Schematic of chemical weathering and dissolved carbon dynamics in response to changing hydrological conditions in a typical karst catchment. This figure was drawn by software Coredraw X7 (http://www.corel.com/cn/).

Biological CO_2_ discharge, *in situ* biodegradation and photosynthesis is the primary driver of the *p*CO_2_ in water^26^,^30^. Because of the relative low value of DOC and few amounts of aquatic plants in valley type of river channel, the effect of biodegradation and photosynthesis to *p*CO_2_ in the Wujiang river could be neglected. Therefore, biological CO_2_ discharge should be the main control on *p*CO_2_, especially during the flooding stage. The values of *p*CO_2_ show a negative correlation with discharge (Fig. 4c) and a power-law dilution effect with increasing discharge. The *p*CO_2_ values exhibit strong chemostatic behavior with respect to increasing discharge when the discharge is greater than 1500 m^3^/s. Biological CO_2_ produced by microbiologic activities and plant respiration would increase under high temperature conditions in summer at Southwest China^26^. So biological CO_2_ discharge is likely responsible for the chemostatic behavior. At extreme discharge rates, the fluid follows near-surface flow paths rather than deep flow paths, and biological CO_2_ does not have enough time to react with rocks. Therefore, biological CO_2_ can be transported to the river directly, which counteracts the dilution effect following periods of high discharge (Fig. 5). Li *et al*.^26^ showed that soil CO_2_ plays an important role in shifting δ^13^C_DIC_ values in a small karst catchment. Epikarst aquifers water, with more negative δ^13^C_DIC_ values than that in riverine water, have an important impact on karstic water carbon in carbonate-rich areas due to the active exchange between the surface water and subsurface flow water. Thus, the water stored in the matrix porosity and soil water with high contents of biological CO_2_ would flow into the river induced by high discharge, leading to decrease of δ^13^C_DIC_ in the Wujiang River. As indicated in Fig. 4d, there is a generally negative correlation between δ^13^C_DIC_ values and discharge. The values of δ^13^C_DIC_ in Wujiang River changing with hydrological variability could not be interpreted in terms of simple dilution. Chemical weathering enhanced by increasing discharge produces large amounts of DIC, which is more positive than those of biological CO_2_. Thus, the δ^13^C_DIC_ does not respond dramatically to the increasing discharge. As seen in Fig. 4d, the δ^13^C_DIC_ values exhibit a power-law mixture effect with increasing discharge, indicating that there is a power-law relationship between the δ^13^C_DIC_ values and discharge, as follows:


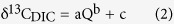


where a, b and c are constants. Usually, c is equal to the δ^13^C value of biological CO_2_. The exponent in the power-law relationship depends on the amount of biological CO_2_. A power law exponent of zero indicates that dilution will not change the values of δ^13^C_DIC_ and that biological CO_2_ will not be directly discharged into the river under high discharge conditions. A power law exponent close to −1 means that a large amount of biological CO_2_ will be discharged into the river, shifting the δ^13^C_DIC_ values.

The δ^13^C_DIC_ values increase with increased (Na* + K)/Ca ratios, which represent the relative contribution of silicate weathering versus carbonate weathering (Fig. S4a). The results indicate that silicate weathering is not responsible for the lower values of δ^13^C_DIC_ in the high-flow season due to relative low (Na* + K)/Ca ratios. Thus, more soil CO_2_ dissolution following rain water discharge would drop δ^13^C-DIC value in the river and counteract the riverine *p*CO_2_ at the same time in the high-flow season. There is a positive relationship between δ^13^C_DIC_ values and SO_4_/DIC ratios (Fig. S4b), suggesting that the lower values of δ^13^C_DIC_ are ascribed to the soil CO_2_ discharge and the reduced ratio of carbonate weathering by H_2_SO_4_. In this study, the power law exponent of δ^13^C_DIC_ versus discharge is close to −0.1, indicating that relative high carbonate weathering rate and biological CO_2_ contribution are stimulated by high discharge likely occurs.

### DIC sources

The sources of DIC can be constrained by δ^13^C_DIC_ values due to the large difference between biological carbon and geological carbon^1^,^31^,^41^. As *p*CO_2_ in riverine water is much higher than *p*CO_2_ in the atmosphere (Fig. 4c), the contribution of atmospheric CO_2_ to DIC can be neglected^5^,^25^,^30^,^32^,^49^,^50^. Thus, the riverine DIC has two major sources: geological source and biological source. As discussed by Telmer and Veizer^32^, carbon isotopes in marine limestones and dolostones deposited since the end of the Proterozoic show typical marine values close to 0‰. C3 plants dominate the study area^25^ with the average δ^13^C value of −27‰. Cerling *et al*.^51^ have reported that there is carbon isotope fractionation of approximately 4.4‰ during the diffusion of CO_2_. Therefore, the carbon isotope of soil CO_2_ in the Wujiang river basin is likely −22.6‰. Given the different δ^13^C_DIC_ of these two endmembers, the source contribution to riverine DIC can be calculated as follows:


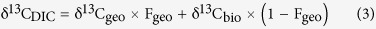


where δ^13^C_geo_ and δ^13^C_bio_ is the δ^13^C values of geological carbon and biological carbon, respectively, and F_geo_ is the proportion of carbon from the geological source. In the case that the DIC is affected by carbonate precipitation and CO_2_ degassing, the F_geo_ is overestimated because of the isotope fractionation^52^.

**Figure 6:**
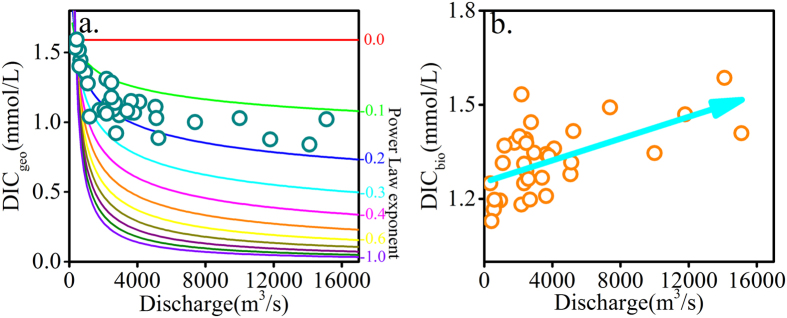
(**a**) Correlation between DIC_geo_ and discharge. (**b**) Correlation between DIC_bio_ and discharge.

The contribution of DIC_bio_ (DIC from biological sources) to DIC in river water increases from 41.5% to 65.4% based on the calculation (Eq. 3). Furthermore, the concentrations of DIC_geo_ (DIC from geological sources) and DIC_bio_ can be determined based on the relative proportions of DIC. The DIC_geo_ concentrations show a power-dilution relationship with discharge (Fig. 6a), indicating that DIC_geo_ exhibits strong chemostatic behavior with respect to discharge changes. Increasing carbonate weathering rates are likely responsible for this chemostatic situation. In contrast, DIC_bio_ values show a positive relationship with discharge (Fig. 6b), suggesting that biological DIC influx is the main driver of the chemostic behavior of total DIC with increasing discharge. Atmospheric precipitation infiltrates into the soil and flush soil CO_2_ into the river. Therefore, high discharge brings excessive biological DIC into the river, leading to increasing DIC_bio_ concentrations with increasing discharge.

Fluvial DIC concentrations and δ^13^C_DIC_ values primarily reflect the mixing of compositionally distinct endmembers (Fig. 6a and b). Physical and biological processed take turns to alter the composition of the DIC pool during changing hydrological conditions (Fig. 5). δ^13^C_DIC_ may be more sensitive than DIC concentrations to hydrological changes (Fig. 4a and d), which is in agreement with Waldran *et al*.^53^. Clearly, physical and biological processes affect DIC concentrations during changing hydrological conditions. Therefore, continuous high-frequency monitoring during field programs should be conducted^53^. The results suggest that δ^13^C_DIC_ values can be useful for revealing the response of biogeochemical processes to riverine hydrological conditions.

## Methods

### The study area

The Wujiang River basin (Fig. S5) is located in the center of the Southeast Asian Karst Region, the largest karst area in the world^8^,^25^. The Wujiang River is the largest tributary on the south bank of the upper Changjiang River, which is the 3^rd^ longest river in the world^8^,^9^. The drainage area is 87 920 km^2^, and the region features a warm subtropical climate^9^. The mean annual precipitation for the last several years has ranged from 850 to 1600 mm^8^, and the occurrence of a seasonal monsoon results in high precipitation during summer and low precipitation during winter^10^. Carbonate rocks are widely exposed in this area, with no significant outcrops of evaporites^8^.

### Sampling and analyses

The sampling site is located at the outlet of the Wujiang River (Fig. S5), where is 45 km away from the mainstream of the Changjiang River. The water samples for chemical and isotopic analyses were collected, monthly, from November 2013 to October 2014, i.e., throughout one entire hydrological year. Additional samples were collected during high-discharge periods. Samples were collected in the middle of the river by boat. pH, teeasured in the field. Alkalinity was determined with 0.02 μM hydrochloric acid mperature(T) and Ec were mwithin 24 hours. The samples were filtered through 0.45 μM cellulose-acetate filter paper. Major cations (K^+^, Na^+^, Ca^2+^ and Mg^2+^) and Si were acidified to pH = 2 with ultrapurified HNO_3_ and measured via Inductively Couples Plasma-optical Emission Spectrometry (ICP-OES) (with an error of 3%). Anions (SO_4_^2−^, Cl^−^ and NO_3_^−^) were analyzed using a Diones ICS90 (with an error of 5%). The DOC was measured using an OI Analytical Aurora 1030 TOC analyzer. For the δ^13^C_DIC_ analysis, the method of Li *et al*.^25^ was used, with a precision of 0.2%. All these analyses were conducted at the State Key Laboratory of Environmental Geochemistry (Institute of Geochemistry, Chinese Academy of Sciences). Daily water discharge data were obtained online from the Ministry of Water Resources (http://www.hydroinfo.gov.cn/). Finally, *p*CO_2_, SIc and all DIC species were calculated based on mass action relationships and the relative equilibrium constants.

## Additional Information

**How to cite this article**: Zhong, J. *et al*. Sensitivity of chemical weathering and dissolved carbon dynamics to hydrological conditions in a typical karst river. *Sci. Rep.* **7**, 42944; doi: 10.1038/srep42944 (2017).

**Publisher's note:** Springer Nature remains neutral with regard to jurisdictional claims in published maps and institutional affiliations.

## Supplementary Material

Supplementary Information
