# Supplementary material for: Sensitivity of chemical weathering and dissolved carbon dynamics to hydrological conditions in a typical karst river
Source: Sci Rep. 2017 Feb 21;7:42944. doi: 10.1038/srep42944 (PMC5318914; doi:10.1038/srep42944)
Supplement: Supplementary Information [file srep42944-s1.pdf]

Supplementary Information *for*

Sensitivity of chemical weathering and dissolved carbon dynamics to hydrological conditions in a typical karst river

Authors: Jun Zhong, Si-liang Li*, Faxiang Tao, Fujun Yue, & Cong-Qiang Liu

*Si-Liang Li (siliang.li@tju.edu.cn)

Contents:

1. Supplementary tables
    1.1. Supplementary Table S1
    1.2. Supplementary Table S2
2. Supplementary figures
    2.1. Supplementary Figures S1
    2.2. Supplementary Figures S2
    2.3. Supplementary Figures S3
    2.4. Supplementary Figures S4
    2.5. Supplementary Figures S5
3. Supplementary Equations
4. Supplementary References

1. Supplementary Tables:

1.1 Supplementary Table S1

Sample Number	Time	EC (μS/cm)	pH	Alk (mmol/L)	Cl^- (ppm)	NO_3^- (ppm）	SO_4^{2-} (ppm)	Ca^{2+} (ppm)	K^+ (ppm)	Mg^{2+} (ppm)	Na^+ (ppm)	SiO_2 (ppm)	TDS (mg/L)	NICB (100%)
W1	11/23/13	365	7.93	2.49	8.9	9.8	60.6	62.9	1.7	9.5	7.4	4.5	306	1.9
W2	12/24/13	366	7.98	2.58	8.1	13.4	60.1	62.6	2.0	10.7	7.7	4.8	312	1.8
W3	01/15/14	397	7.99	2.62	7.8	12.5	66.0	63.9	2.2	11.8	7.5	4.8	323	1.9
W4	02/17/14	407	8.07	2.74	10.4	13.5	66.6	70.1	1.9	12.1	8.3	4.2	339	3.2
W5	03/25/13	380	8.05	2.67	11.1	15.1	80.1	71.0	2.5	13.9	10.8	4.7	356	3.6
W6	04/18/14	340	8.09	2.46	8.5	0.7	45.2	53.3	1.6	9.8	6.5	5.0	278	2.4
W7	05/22/14	351	8.13	2.54	5.5	9.6	50.2	57.0	1.8	10.8	5.1	5.5	289	1.9
W8	06/19/14	385	8.13	2.48	5.6	6.7	50.3	58.2	1.9	10.6	5.1	5.5	287	3.9
W9	06/21/14	381	8.18	2.48	4.5	9.1	52.3	57.2	2.0	12.5	3.3	6.3	287	3.6
W10	06/22/14	357	8.14	2.39	4.6	12.0	43.3	56.0	1.8	9.9	4.0	5.4	269	3.5
W11	06/23/14	364	8.15	2.37	4.5	11.4	40.0	52.3	1.6	9.5	3.7	5.2	260	1.8
W12	06/24/14	345	8.17	2.38	5.0	7.4	45.5	56.5	1.8	10.1	4.6	5.1	272	4.8
W13	06/26/14	384	8.16	2.36	4.7	10.9	40.2	52.2	1.5	9.9	3.7	5.2	260	2.2
W14	06/29/14	354	8.15	2.44	5.2	2.6	42.9	54.7	1.7	9.7	4.4	4.9	271	3.9
W15	07/02/14	344	8.15	2.46	4.9	2.7	41.7	54.2	1.7	9.3	4.3	5.2	269	3.3
W16	07/03/14	336	8.20	2.35	4.2	5.3	38.4	53.5	1.4	9.0	3.2	5.0	256	4.1
W17	07/04/14	341	8.25	2.48	4.3	8.0	39.0	53.7	1.6	8.9	3.5	5.2	265	1.7
W18	07/05/14	344	8.26	2.38	4.1	10.1	36.6	49.3	1.4	8.4	3.3	5.0	251	-0.4
W19	07/06/14	357	8.26	2.39	4.7	11.4	42.7	55.1	1.7	9.6	4.0	5.1	266	2.0
W20	07/07/14	358	8.30	2.35	4.7	8.5	43.4	53.3	1.7	10.0	4.0	5.0	263	3.3
W21	07/12/14	350	8.28	2.40	4.7	3.1	41.5	52.0	1.6	9.4	3.8	5.1	262	2.6
W22	07/13/14	342	8.24	2.35	4.3	10.6	38.7	51.4	1.6	8.8	3.6	5.2	254	1.3
W23	07/14/14	332	8.19	2.31	3.6	6.6	32.1	46.4	1.5	8.0	3.1	4.7	239	0
W24	07/15/14	315	8.20	2.33	3.5	10.2	33.5	51.3	1.5	8.2	3.4	5.4	247	2.7
W25	07/16/14	323	8.22	2.36	3.8	7.7	35.5	50.6	1.5	8.6	2.9	5.3	250	1.8
W26	07/17/14	355	8.22	2.41	4.1	6.5	38.4	52.4	1.5	9.2	3.2	5.4	259	2.4
W27	07/18/14	361	8.21	2.41	4.5	11.5	46.2	52.5	1.5	9.7	4.0	5.3	268	0
W28	07/19/14	340	8.21	2.33	4.2	11.9	43.1	53.0	1.4	9.5	3.7	5.5	261	2.1
W29	07/20/14	342	8.27	2.29	4.2	11.4	43.0	52.9	1.7	9.4	3.5	5.5	258	2.4
W30	07/21/14	335	8.19	2.33	4.1	3.0	42.6	52.5	1.7	9.4	3.5	5.7	259	3.6
W31	08/12/14	388	8.19	2.54	5.4	13.2	55.8	56.7	2.1	11.5	5.1	4.8	294	0.6
W32	09/13/14	345	8.19	2.59	3.9	11.7	49.7	55.0	1.9	10.0	4.3	5.3	285	-0.7
W33	10/13/14	399	8.16	2.57	4.7	14.6	69.4	58.6	2.4	11.2	6.0	4.9	312	-2.0
W34	10/25/14	326	8.12	2.57	5.2	14.0	65.4	59.4	2.0	10.4	5.5	4.8	307	-1.7

Supplementary Table S1: Full dataset on hydrochemical characteristics on Wujiang River from November 2013 to October 2014. NICB=$(TZ^+-TZ^-)\times100\%/(TZ^++TZ^-)$

1.2 Supplementary Table S2

Sample Number	DOC (ppm)	DIC (mmol/L)	$\delta^{13}C_{DIC}$ (‰)	H_2CO_3 (mmol/L)	HCO_3^- (mmol/L)	CO_3^{2-} （mmol/L）	pCO_2 （μatm）	SIc	F_{DIC} （mol/L）	F_{DOC} (mg/L)	F_{sil} (kg/L)	F_{carb} (kg/L)	DIC_{bio} (mmol/L)	DIC_{geo} (mmol/L)
W1	0.89	2.55	-10.6	0.07	2.47	0.010	1595	0.45	2.38	0.83	9.8	60.1	1.20	1.36
W2	0.98	2.63	-10.2	0.06	2.56	0.014	1619	0.69	1.72	0.64	8.3	42.4	1.19	1.45
W3	1.05	2.68	-9.8	0.07	2.60	0.011	1372	0.54	1.52	0.60	7.4	38.0	1.17	1.52
W4	1.28	2.78	-10.1	0.05	2.71	0.018	1403	0.84	0.98	0.45	3.7	26.1	1.25	1.53
W5	1.32	2.72	-9.4	0.06	2.65	0.014	1215	0.65	1.17	0.56	6.5	32.3	1.13	1.59
W6	1.02	2.49	-10.7	0.04	2.43	0.017	1202	0.72	5.43	2.23	21.7	121	1.18	1.31
W7	1.15	2.57	-11.3	0.04	2.51	0.017	1025	0.69	6.36	2.84	28.9	148	1.29	1.28
W8	1.26	2.51	-12.5	0.04	2.45	0.019	1096	0.79	6.12	3.08	28.7	148	1.39	1.12
W9	1.17	2.51	-12.3	0.04	2.45	0.020	1024	0.81	10.3	4.81	46.3	255	1.36	1.15
W10	1.01	2.41	-12.6	0.04	2.36	0.018	1031	0.78	8.78	3.68	38.9	212	1.34	1.07
W11	0.97	2.40	-12.7	0.04	2.34	0.018	1000	0.76	7.02	2.85	29.0	159	1.35	1.05
W12	0.97	2.40	-12.3	0.04	2.35	0.019	969	0.80	5.62	2.28	25.4	138	1.31	1.09
W13	1.02	2.39	-11.8	0.04	2.33	0.017	903	0.69	5.59	2.38	22.3	128	1.25	1.14
W14	0.94	2.47	-12.6	0.04	2.41	0.019	1050	0.78	4.37	1.66	17.9	101	1.38	1.09
W15	1.03	2.49	-12.7	0.04	2.43	0.019	1058	0.78	5.12	2.12	21.6	115	1.40	1.09
W16	1.03	2.37	-13.8	0.03	2.31	0.020	885	0.81	6.51	2.84	24.2	152	1.44	0.92
W17	1.01	2.49	-13.5	0.03	2.44	0.024	831	0.89	18.4	7.46	71.8	408	1.49	1.00
W18	0.97	2.39	-12.1	0.03	2.34	0.023	791	0.84	12.1	4.89	45.5	256	1.28	1.11
W19	1.04	2.40	-12.6	0.03	2.35	0.024	791	0.88	9.14	3.94	38.9	217	1.34	1.07
W20	1.05	2.36	-12.1	0.03	2.30	0.025	711	0.90	6.10	2.72	26.0	144	1.26	1.09
W21	1.04	2.41	-12.8	0.03	2.36	0.025	762	0.88	2.87	1.24	11.4	64.3	1.37	1.04
W22	1.05	2.36	-11.6	0.03	2.31	0.022	815	0.83	8.57	3.80	35.5	192	1.21	1.15
W23	0.97	2.34	-11.6	0.03	2.28	0.018	860	0.69	6.26	2.59	24.0	127	1.20	1.14
W24	0.94	2.35	-12.7	0.03	2.29	0.019	843	0.74	12.0	4.81	52.3	267	1.32	1.03
W25	1.04	2.38	-12.8	0.03	2.32	0.021	848	0.81	23.8	10.4	91.3	521	1.35	1.03
W26	1.08	2.43	-13.1	0.03	2.38	0.022	880	0.83	36.7	16.4	142	822	1.41	1.02
W27	1.14	2.43	-14.8	0.03	2.37	0.021	898	0.82	34.2	16.1	140	773	1.59	0.84
W28	1.17	2.35	-14.2	0.03	2.30	0.020	865	0.81	27.7	13.8	119	652	1.47	0.88
W29	1.13	2.30	-13.9	0.03	2.25	0.023	733	0.86	12.0	5.93	53.9	286	1.42	0.89
W30	1.22	2.35	-12.2	0.03	2.30	0.018	862	0.73	7.97	4.14	35.5	184	1.27	1.08
W31	1.13	2.56	-12.2	0.04	2.50	0.021	949	0.83	6.32	2.80	28.8	148	1.38	1.18
W32	0.99	2.60	-13.4	0.03	2.54	0.027	768	0.93	5.68	2.17	25.9	125	1.53	1.06
W33	1.04	2.59	-11.5	0.04	2.53	0.021	1059	0.83	2.83	1.14	15.4	66.1	1.31	1.28
W34	0.90	2.60	-10.4	0.04	2.54	0.018	1118	0.77	1.55	0.54	7.2	36.7	1.20	1.40

Supplementary Table S2: Full dataset on dissolved carbon characteristics, chemical weathering rates and DIC sources on Wujiang River from November 2013 to October 2014.

2. Figures:

2.1. Supplementary figure S1

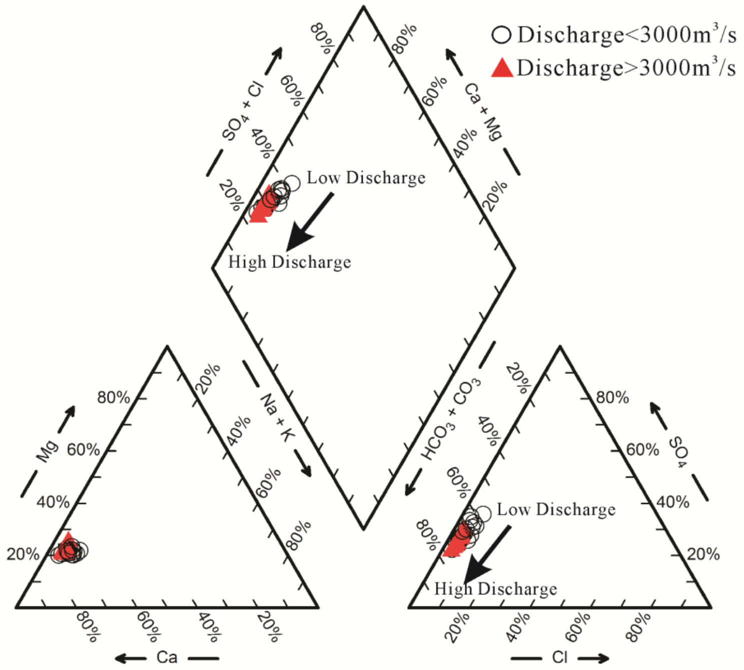

Figure S1. Piper diagram for surface water samples collected in Wujiang River. Figure was produced using AqQa (https://www.rockware.com/product/overview.php?id=150).

2.2. Supplementary figure S2

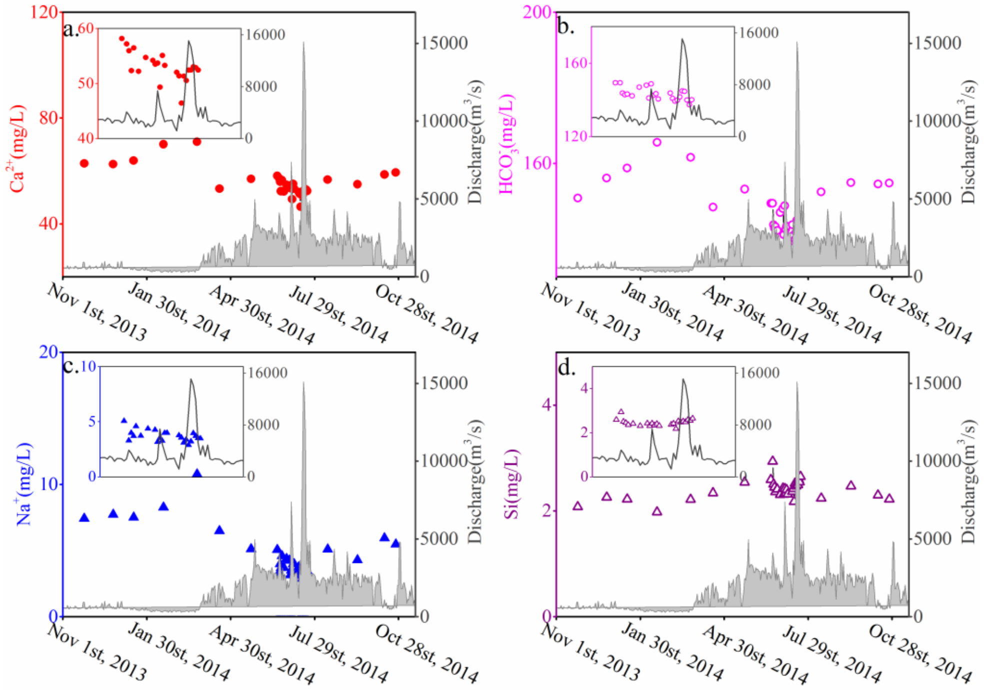

Figure S2. Temporal variations of daily discharge (solid line) and concentrations of Ca^{2+}, HCO_3^-, Na^+ and SiO_2 for the Wujiang River between November 2013 and October 2014, and detailed picture showing the temporal variations in the monsoon season.

2.3. Supplementary figure S3

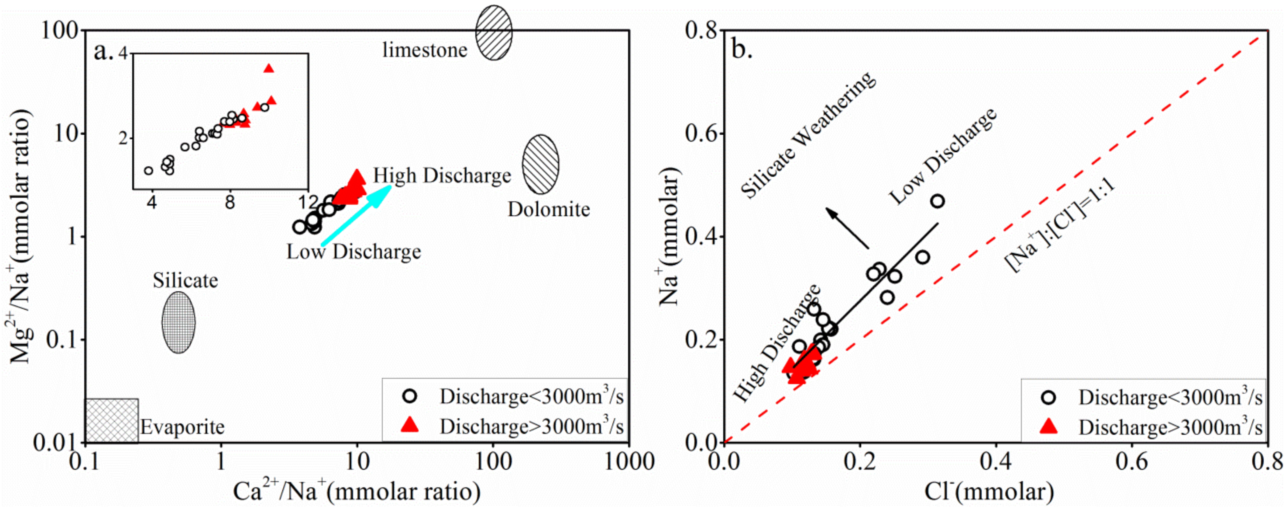

Figure S3. a. Plots of Mg^{2+}/Na^{+} versus Ca^{2+}/Na^{+} ratios for the Wujiang River. The endmembers are from Han and Liu[1]. b. Na^{+} plots against Cl^{-}.

2.4. Supplementary figure S4

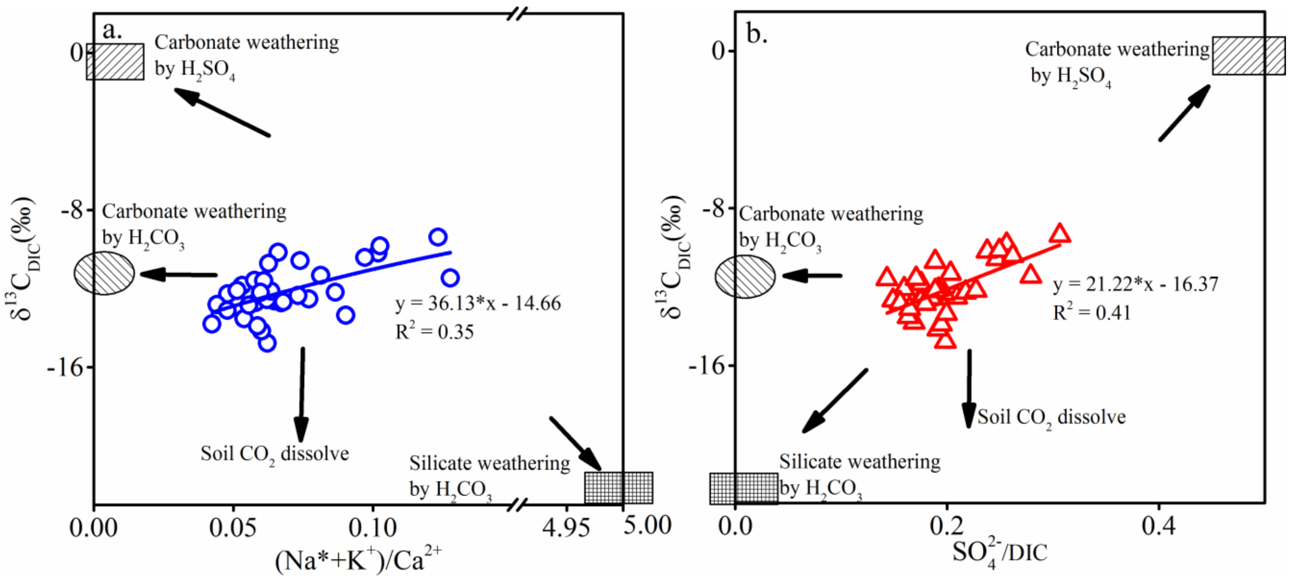

Figure S4. a Correlation between $(Na^{*}+K^{+})/Ca^{2+}$ and $\delta^{13}C_{DIC}$. b. Correlation between SO_4^{2-}/DIC and discharge.

2.5. Supplementary figure S5

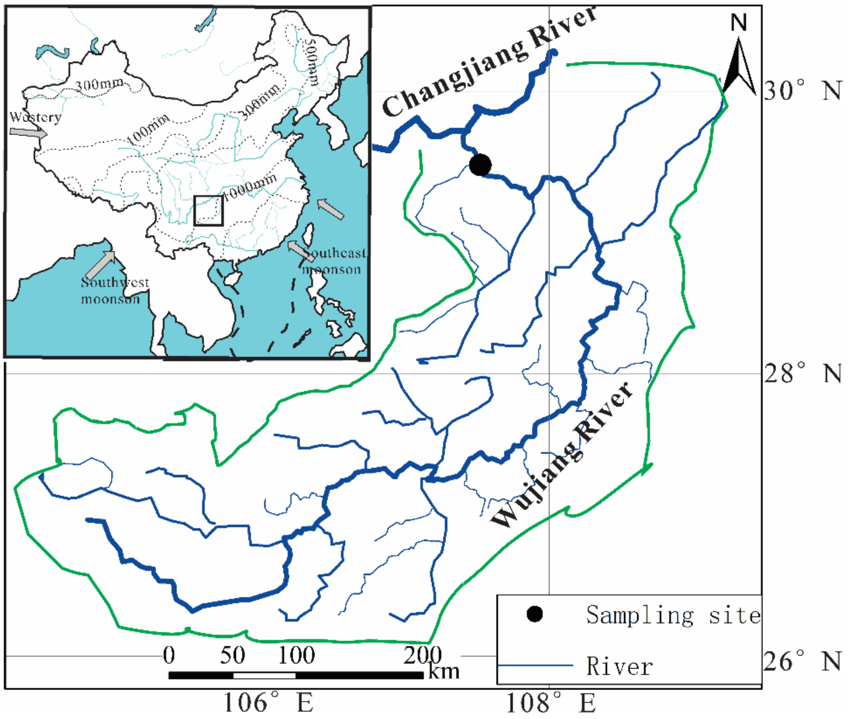

Figure S5. Map showing the sampling site of the Wujiang River. This figure was drawn by software Coredraw X7 (http://www.corel.com/cn/).

3. Equations

The local CO_2 partial pressure (pCO_2) can be estimated by taking into account the effect of elevation[3,4]:

$P_Z=P_0\ \exp(-Z/H)$ (S1)

Forward model:

The mass balance equation in the forward model for element X in riverine water can be expressed as follows Han and Liu[1], Li, et al.[5], Galy[6] and Fan, et al.[7] .

$[X]_{river} = [X]_{atmosphere} + [X]_{carbonate} + [X]_{silicate} + [X]_{anthropogenic}$ (S2)

$[Cl^-]_{atmosphere} = 0.027$ mmol/L (S3)

$[Cl^-]_{river} = [Cl^-]_{atmosphere} + [Cl^-]_{anthropogenic}$ (S4)

$[Na^+]_{river} = [Cl^-]_{river} + [Na^+]_{silicate}$ (S5)

$[K^+]_{river} = [K^+]_{silicate}$ (S6)

$[Ca^{2+}]_{river} = [Ca^{2+}]_{carbonate} + [Ca^{2+}]_{silicate}$ (S7)

$[Mg^{2+}]_{river} = [Mg^{2+}]_{carbonate} + [Mg^{2+}]_{silicate}$ (S8)

$[Mg/K]_{silicate} = 2$ (S9)

$[Ca/Na]_{silicate} = 0.2$ (S10)

$F_{Carb} = ([Ca^{2+}]_{Carb}\times M_{Ca}+[Mg^{2+}]_{Carb}\times M_{Mg})\times Discharge$ (S11)

$F_{Sil} = ([Ca^{2+}]_{Sil}\times M_{Ca}+[Mg^{2+}]_{Sil}\times M_{Mg}+[Na^+]_{Sil}\times M_{Na}+[K^+]_{Sil}\times M_K+[Si]\ \times M_{SiO2})\times Discharge$ (S12)

Where M refers to the molar mass, the subscript Carb, Sil represents major ions of carbonate and silicate weathering sources, respectively.

4. References:

1 Han, G. & Liu, C.-Q. Water geochemistry controlled by carbonate dissolution: a study of the river waters draining karst-dominated terrain, Guizhou Province, China. *Chemical Geology* **204**, 1-21, doi:10.1016/j.chemgeo.2003.09.009 (2004).

2 Wu, L., Huh, Y., Qin, J., Du, G. & van Der Lee, S. Chemical weathering in the Upper Huang He (Yellow River) draining the eastern Qinghai-Tibet Plateau. *Geochimica et Cosmochimica Acta* **69**, 5279-5294, doi:10.1016/j.gca.2005.07.001 (2005).

3 Moon, S., Huh, Y., Qin, J. & Vanpho, N. Chemical weathering in the Hong (Red) River basin: Rates of silicate weathering and their controlling factors. *Geochimica et Cosmochimica Acta* **71**, 1411-1430, doi:10.1016/j.gca.2006.12.004 (2007).

4 Li, S. *et al.* Major element chemistry in the upper Yangtze River: A case study of the Longchuanjiang River. *Geomorphology* **129**, 29-42, doi:10.1016/j.geomorph.2011.01.010 (2011).

5 Galy, A. F.-L., C. Weathering processes in the Ganges–Brahmaputra basin and the riverine alkalinity budget. *Chemical Geology* **159** (1999).

6 Fan, B.-L. *et al.* Characteristics of carbonate, evaporite and silicate weathering in Huanghe River basin: A comparison among the upstream, midstream and downstream. *Journal of Asian Earth Sciences* **96**, 17-26, doi:10.1016/j.jseaes.2014.09.005 (2014).
